# Identifying Prenatal Alcohol Exposure and Children Affected by It: A Review of Biomarkers and Screening Tools

**DOI:** 10.35946/arcr.v43.1.03

**Published:** 2023-05-25

**Authors:** Julie A. Kable, Kenneth Lyons Jones

**Affiliations:** 1 Department of Psychiatry and Behavioral Sciences, Emory University School of Medicine, Atlanta, Georgia; 2 Department of Pediatrics, Emory University School of Medicine, Atlanta, Georgia; 3 Department of Pediatrics, University of California San Diego, La Jolla, California

**Keywords:** alcohol, prenatal alcohol, FASD, identification, biomarkers, fetal alcohol spectrum disorders, prenatal exposure delayed effects

## Abstract

**PURPOSE:**

Early identification of prenatal alcohol exposure (PAE) and of those in need of services resulting from this exposure is an important public health concern. This study reviewed the existing literature on potential biomarkers and screening tools of PAE and its impact.

**SEARCH METHODS:**

Electronic databases were searched for articles published between January 1, 1996, and November 30, 2021, using the following search terms: (“fetal alcohol” or “prenatal alcohol” or “FASD” or “alcohol-related neurodevelopmental disorder” or “ARND” or “ND-PAE”) and (“screening” or “identification” or “biomarker”). Duplicate articles were electronically eliminated. Titles and abstracts were reviewed for appropriateness, and selected articles were retrieved for further analysis. Additional articles were added that were referenced in the reviewed articles or identified from expert knowledge. Information about the characteristics of the sample, the biomarker or screening tool, and the predictive validity outcome data were abstracted. A narrative analysis of the studies was then performed on the data.

**SEARCH RESULTS:**

A total of 3,813 articles were initially identified, and 1,215 were removed as duplicates. Of the remaining articles, 182 were identified as being within the scope of the review based on title and abstract inspection, and 181 articles were successfully retrieved. Of these, additional articles were removed because they were preclinical (3), were descriptive only (13), included only self-report of PAE (42), included only mean group comparison (17), were additional duplicates (2), focused on cost analysis (9), missed predictive validity data (24), or for other reasons (23). The remaining articles (*n* = 48) were abstracted. An additional 13 manuscripts were identified from these articles, and two more from expert knowledge. A total of 63 articles contributed to the review.

**DISCUSSION AND CONCLUSIONS:**

Biomarkers and screening tools of PAE and its impact fall short of ideal predictive validity characteristics. Higher specificity than sensitivity was found for many of the biomarkers and screening tools used to identify PAE and its impact, suggesting that current methods continue to under-identify the full range of individuals impacted by PAE. Exceptions to this were found in recent investigations using microRNAs related to growth and vascular development, proteomic changes associated with PAE, and combinations of markers estimating levels of various cytokines. Replications of these findings are needed across other samples to confirm the limited data available. Future research on biomarkers and screening tools should attend to feasibility and scalability of implementation. This article also recommends a systematic process of evaluation to improve early identification of individuals impacted by PAE so that harm reduction and habilitative care efforts can be implemented.

Although the awareness of the negative impact of prenatal alcohol exposure (PAE) was already alluded to in ancient writings^1^ and the impact of ethanol embryopathy in animal models was studied as early as 1910,^2^ the conceptualization of a syndrome associated with PAE was not recognized within modern medicine until the mid-20th century.^3^,^4^ The syndrome or disorder was not uniformly accepted, however, and debates occurred within the field related to the operationalization of criteria for making a clinical diagnosis. In 1996, a group of scientists were brought together under the auspices of the Institute of Medicine (IOM) to delineate criteria for a diagnosis and a public health care plan for addressing the needs associated with the condition.^5^ This committee established the first consensus criteria for fetal alcohol syndrome (FAS) and recognized associated conditions, such as partial FAS (pFAS), alcohol-related birth defects (ARBD), and alcohol-related neurodevelopmental disorder (ARND). Various operational definitions of the IOM report’s diagnostic guidelines have been used to make a clinical diagnosis.^6^–^17^ In all cases, these diagnostic formulations struggle with identifying infants negatively impacted by PAE because few tools are available for assessing early brain development. In addition, many of the diagnostic formulations require input from complex medical teams evaluating different domains of impact, which are costly and heavily constrained by the number of professionals qualified to carry out the assessments.

Estimates of the prevalence of prenatal alcohol-related disorders have varied dramatically over the years. In the initial IOM report, which reviewed several registries and clinic-based studies, the estimate of FAS was reported to be in the range of 0.5 to 3 cases per 1,000 births;^5^ however, more recent estimates have been much higher. A large consortium that estimated the prevalence of fetal alcohol spectrum disorders (FASD)—an umbrella term used to refer to a range of conditions (FAS, pFAS, ARBD, and ARND) associated with PAE—in four communities within the United States using active case ascertainment yielded a conservative estimate of 11.3 to 50 per 1,000 births^18^ and an even higher weighted prevalence estimate of 31 to 99 per 1,000 births. A review of more than 24 unique studies carried out throughout the world resulted in a prevalence estimate of 8 per 1,000 births with a 95% confidence interval of 5 to 12 per 1,000 births.^19^ Variations in the estimates are likely related to differences in diagnostic criteria used to estimate the prevalence of the disorder across studies, use of active versus passive surveillance methods, and regional variations in drinking patterns. Historically, documentation of PAE has been difficult to obtain due to unreliability of the self-report of women drinking in pregnancy and potential social stigma associated with acknowledging alcohol use in pregnancy that can result in underreporting of PAE.^20^ The lack of recognition by various health professionals for the cluster of symptoms associated with the diagnosis of FASD also has contributed to under-recognition of those impacted by PAE.^21^

In anticipation of this problem, the IOM report outlined the need for biological markers of alcohol teratogenesis to help with resolving variations in case definitions.^5^ The term “biomarker” refers to a broad collection of medical signs that can be used to identify a disease and can be measured accurately and reliably.^22^ Biomarkers differ from medical symptoms, which are collected via patient report of their status and typically refer to biological measurements associated with the disease state. Biomarkers have the advantage of reducing ambiguity in patient reporting of symptoms but are only useful if they can validly predict a clinical endpoint—that is, if they can appropriately identify the disease state and avoid misclassification of individuals who do not have the condition. In the case of PAE, the clinical endpoint may be the identification of an alcohol-exposed pregnancy or of those negatively impacted by their exposure. Ideally, the identification would occur as early as possible during or after pregnancy to enhance opportunities for intervention. Identification during pregnancy could lead to harm reduction efforts, whereas early postnatal recognition of infants negatively impacted by PAE would increase the opportunities for access to habilitative care to optimize early brain development during phases of high neuroplasticity.^23^ In addition to biomarkers, screening tools that sample symptoms of the disease state, or some combination of these, may be useful in identifying those negatively impacted by PAE. The development of innovative methods and tools that can be used to reduce the costly diagnostic assessment burden that constrains the identification of individuals in need of services are of particular value as such tools would allow for improved scalability and implementation in resource-poor areas of the world.

This review attempts to clarify potential advancements in the identification of biomarkers of PAE or its impact that could be used to improve early recognition of those adversely affected since the original IOM report’s call for the development of biomarkers of alcohol-related teratogenesis. To this end, the authors conducted a review of the literature on the predictive validity of biomarkers or screening tools for identification of PAE or FASD and performed a narrative analysis of the findings.

## Search Methods

Studies were considered for review if the article was published or available online between January 1, 1996, the first day of the IOM report publication year, and November 30, 2021. The target population consisted of individuals of any age who had been diagnosed with PAE or with a clinical disorder associated with PAE (i.e., FAS, pFAS, ARND, ARBD, and neurobehavioral disorder associated with prenatal alcohol exposure [ND-PAE]).^24^

In addition, the article’s focus had to include screening or identification of PAE or one of the clinical disorders associated with PAE. The article also had to include empirical data related to the screening or identification procedures and provide some aspect of the biomarker’s predictive characteristics. Predictive validity characteristics evaluated in each study included sensitivity, specificity, positive predictive value (PPV), negative predictive value (NPV), accuracy, and area under the curve (AUC). Sensitivity refers to the probability that the test is positive when the condition is present. Specificity refers to the probability that the test is negative when the condition is not present. PPV refers to the probability that the condition is present when the test is positive. NPV refers to the probability that the condition is not present when the test is negative. Accuracy refers to the overall probability that the case is correctly classified from the test. Criterion descriptors for the predictive values are as follows: 90–100%, Excellent; 80–89%, Good; 70–79%, Fair; and below 70%, Poor. Finally, AUC is derived from creating receiver operating curves by plotting the true positive rate (sensitivity) relative to the false positive rate (1-specificity). The AUC references the area on the graph created by the regression line relative to the chance rate of prediction. Values of 1 would indicate perfect condition, and values of 0.50 would indicate chance prediction using a binary (yes/no) model.

**Figure 1:**
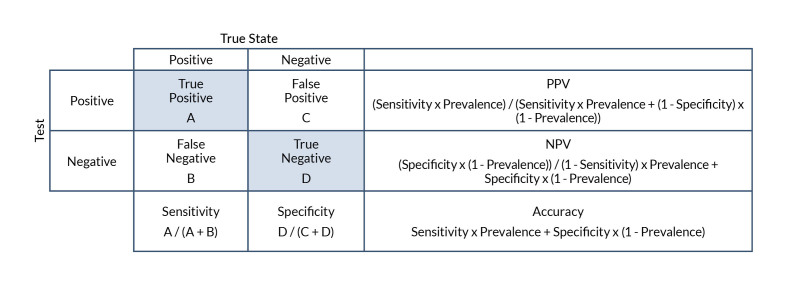
Confusion matrix The confusion matrix provides definitions of the various predictive validity terms within a contingency table where cases are plotted relative to the prediction variable and the designated “true state.” True state refers to whether the individual has a disease or condition (positive) or does not have a disease or condition (negative), and the test reflects the outcome of the criterion used to indicate a positive or negative prediction of disease state. Sensitivity refers to the probability that the test is positive when the condition is present. Specificity refers to the probability that the test is negative when the condition is not present. PPV refers to the probability that the condition is present when the test is positive. NPV refers to the probability that the condition is not present when the test is negative. Accuracy refers to the overall probability that the case is correctly classified from the test. *Note:* NPV, negative predictive value; PPV, positive predictive value.

Definitions for the first five predictive validity characteristics and formulas for computing them are outlined in Figure 1, a confusion matrix that illustrates the classic prediction modeling used when comparing a test’s ability to identify a given state or condition. The confusion matrix is a contingency table that presents the frequency of individuals categorized across two dimensions, the actual true state of whether or not an individual has a disease or condition, and the predicted state derived from the results of the testing indicating the presence of the disease or not.

To identify studies, the following electronic databases were searched: PsycInfo, PubMed, Medline, Web of Science, ERIC, and the Cochrane Central Register of Control Trials. Search terms used were [“fetal alcohol” or “prenatal alcohol” or “FASD” or “alcohol-related neurodevelopmental disorder” or “ARND” or “ND-PAE”] and [“screening” or “identification” or “biomarker”]. Document type was limited to “articles,” but no language restrictions were placed on the initial search. Despite extensive work in animal models of PAE on various promising biomarkers, only articles using humans were selected as the focus of this study was to analyze the current knowledge of potential tools that could be used to identify people affected by PAE. Preclinical biomarker methodologies still need translation into human populations to effectively evaluate their predictive characteristics.

**Figure 2:**
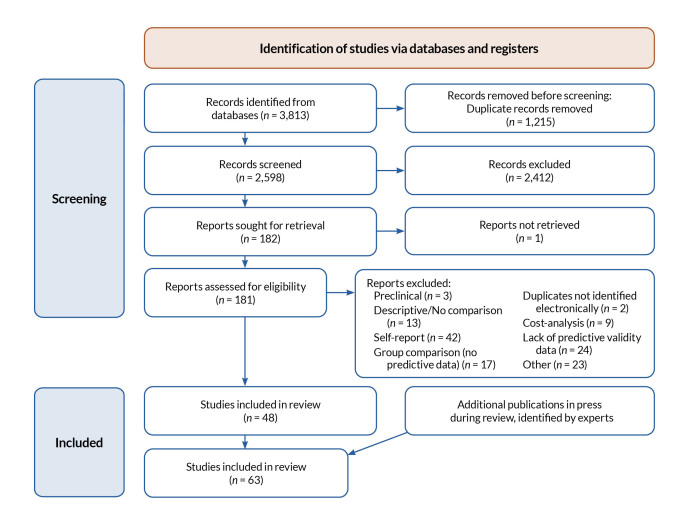
Flow diagram of the steps in the screening process for this review.

References were then merged into Endnote X9.3.1 and screened for duplicates. The remaining studies were then reviewed to eliminate nonempirical studies (i.e., reviews or editorial articles) and those involving training of professionals to screen. Articles were also excluded if they established group differences without analyzing the predictive validity of the outcome or were descriptive of PAE in a given population. While establishing group differences may be a first step in establishing the utility of a biomarker or screening tool, such differences do not establish a tool’s predictive utility. IQ tests are a classic example of tools that consistently demonstrate group differences between PAE groups relative to community samples without exposure;^25^ however, they have little predictive utility when used independently as a result of the wide range of outcomes seen in individuals with PAE and its associated overlap with comparison samples. A flow diagram (Figure 2) outlines the various steps in screening the articles and the number of articles at each step.

## Search Results

A total of 3,813 articles were initially captured by the search, and 1,215 were identified as duplicates. Article titles and abstracts were then screened for inclusion, and an additional 2,412 were eliminated, leaving 181 full articles that were retrieved. One article could not be retrieved. The full articles were reviewed for appropriateness, and 133 articles were excluded for the following reasons: three were preclinical, 13 were descriptive only, 42 related to predictive utility of self-report methods of PAE, 17 were identified as group comparison studies, two were additional duplicates not identified electronically, nine were related to cost analysis, and 24 after further review did not have predictive data. This left 48 articles; however, upon further review, 13 additional articles were identified that were not retrieved by the search. Moreover, two additional articles were identified based on expert knowledge. This resulted in 63 articles included in the review.

Biomarkers and screening tools were categorized as predicting prenatal exposure status or alcohol-related teratogenesis in the offspring. Appendix 1 provides details on the articles that involved biomarker predictors of PAE status, and Appendix 2 provides details on biomarker predictors of FASD and associated symptoms. Both appendices list the articles in alphabetical order by the first author’s last name as many involve the evaluation of several biomarkers and predictors within one study. Appendix 3 provides details on other screening tool predictors of FASD and associated symptoms, including craniofacial features, neurophysiological responses, neuroimaging analyses, questionnaire responses, and various test batteries assessing performance. As typically only one screening tool was evaluated within a study, Appendix 3 groups studies by screening tool category and then lists studies alphabetically.

Predictive validity information was obtained from information explicitly stated in the text or tables or was computed from information regarding cell sizes in the predictive validity tables provided in the article or as described in the text. Computations were performed using MedCalc software for diagnostic test evaluation (MedCalc Software Ltd, Ostend, Belgium). Predictive validity values are presented as percentages with the exception of AUC values, which were reported in proportions of accurate diagnostic classification with values of 0 to 1.00.

**Figure 3:**
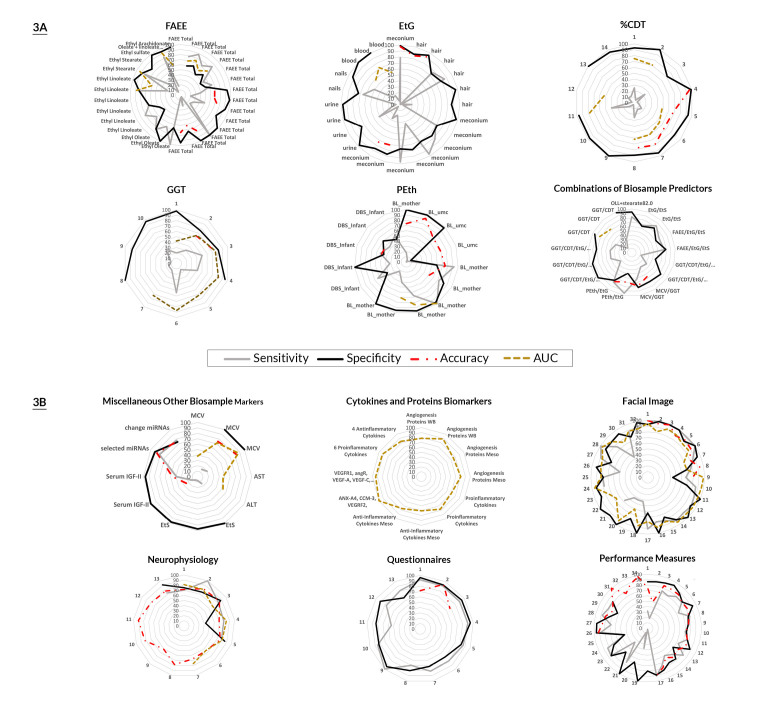
Radial curves of PAE biomarkers (A) and of biomarkers and screening tools for PAE and its impact (B) Radial graphs indicate the specificity (gray curves), sensitivity (black curves), accuracy (red dotted curves), and area under the curve (AUC) values (gold curves) relative to the criterion evaluated in the study. Point estimates or the obtained values of the validity characteristics were provided for each unique outcome of the study. For studies that compared the biomarkers’ response to common outcomes defined differently (e.g., self-report using different assessment tools) only the point estimates reflecting the least and greatest value were included to reflect the range of validity. AUC values were multiplied by 100 to facilitate plotting them on the same curves as the other predictive values. The radial graph plots the various findings along curves with increasing prediction (0–100). Radial curves allow for a quick visual analysis of each of the predictive characteristics for each type of biomarker or screening tool and the variation across the findings. Greater numbers of findings displayed in a graph result in smoothing of the curve. The strength of the prediction is reflected along the radius of the circle so that values in the outer region reflect greater predictive validity and those in the inner region reflect lower levels of predictive validity. Curves with more points along the outer ring with less deviance inward reflect increased predictive status and uniformity in the prediction. Separate colored lines are used to connect the points along with curve for each of the predictive characteristics. Criterion descriptors for the values plotted above are as follows: 90–100, Excellent; 80–89, Good; 70–79, Fair; and below 70, Poor. *Note:* ALT, alanine aminotransferase; angR, angR protein; ANX-A4, annexin-A4; AST, aspartate aminotransferase; AUC, area under the curve; BL, blood level; CCM-3, cerebral cavernous malformation 3 (a protein); CDT, carbohydrate-deficient transferrin; a protein; DBS, dried blood spots; EtG, ethyl glucuronide; EtS, ethyl sulfate; FAEE, fatty acid ethyl esters; GGT, gamma-glutamyltransferase; IGF-II, insulin-like growth factor-II; MCV, mean corpuscular volume; miRNAs, micro RNAs; NPV, negative predictive value; OLL, oleate + linoleate + linolenate; PEth, phosphatidylethanol; PPV, positive predictive value; umc, umbilical cord; VEGF, vascular endothelial growth factor; VEGFR, vascular endothelial growth factor receptor; WB, Western Blotting Procedures.

The sensitivity, specificity, accuracy, and AUC values were plotted on radial curves for each type of biomarker, with each type of predictive characteristic color-coded (see Figure 3). AUC values were multiplied by 100 to facilitate plotting them on the same curves as the other predictive values. The obtained values for each of the validity characteristics were provided for each unique outcome of the study. For studies that compared the biomarker response to common outcomes defined differently (e.g., self-report using different assessment tools), only the obtained values reflecting the least and greatest value were included to reflect the range of validity. Radial curves plot individual values of these predictive parameters along a curve with increasing number of indicators smoothing out until the curve is circular. The strength of the prediction is reflected along the radius of the circle so that values in the outer region reflect increased predictive validity and those in the inner region reflect lower levels of predictive validity. Radial curves allow for a quick visual analysis of each of the predictive characteristics for each type of biomarker or screening tool and the variation across the findings. Curves with more points along the outer ring with less deviance inward reflect increased predictive status and uniformity in the prediction.

### Biomarkers

Biomarkers of PAE were derived from various biological samples obtained from mothers, including blood (plasma and dried blood spots), urine, hair, and fingernail clippings. Sources of biomarkers evaluated in the infant included blood (plasma and dried blood spots) and meconium. Additional biomarkers of PAE or its effects were obtained from placental tissue and the umbilical cord. Biomarkers were evaluated against group status determined from maternal self-report of alcohol consumption and the offspring’s FASD symptomatology or diagnosis.

One group of biomarkers evaluated included fatty acid ethyl esters (FAEE) derived from hair or meconium. FAEE are metabolites of ethanol and provide a long-term estimate of alcohol consumption over the course of a pregnancy. They were analyzed either in a collective grouping of FAEE or individually (i.e., ethyl stearate, ethyl linoleate); in total, 30 obtained values or point estimates of predictive validity were provided across 12 studies.^26^–^37^ In three additional studies, FAEE were used as the outcome to assess other biomarker predictors.^38^–^40^ The radial graph of the predictive characteristics of FAEE in combination or separately (see Figure 3A) suggests that their specificity (range, 43%–100%; median, 83%) as biomarkers is significantly better than their sensitivity (range, 4%–100%; median, 65%); overall accuracy estimates fell in the poor to fair range (range, 62%–79%; median, 68%). Estimates of the AUC values were variable, ranging from poor to excellent (range, 0.52–0.93; median, 0.71). There was no clear pattern that a summation of several FAEE or any one FAEE provided better prediction.

Other biomarkers assessed included gamma-glutamyltransferase (GGT),^35^,^41^–^46^ carbohydrate-deficient transferrin (CDT),^38^,^41^–^46^ ethyl glucuronide (EtG),^30^,^31^,^35^,^38^–^41^,^43^,^44^,^47^–^52^ ethyl sulfate (EtS),^31^,^35^,^41^ and mean corpuscular volume (MCV).^45^ GGT, CDT, and MCV provide an indirect assessment of the impact of heavy and chronic alcohol use on the mother’s metabolic functioning. Estimates of GGT can be obtained from plasma, urine, and hair, whereas CDT and MCV estimates are only obtained from plasma. EtG and EtS are metabolites of ethanol that are present in hair, meconium, urine, and nails. Predictive validity information was found for seven studies using GGT (10 point estimates), seven studies using CDT (13 point estimates), and one study using MCV (three point estimates). Fifteen studies with 24 point estimates were identified for EtG. Three studies evaluated EtS,^31^,^35^,^41^ but only two provide estimates of EtS alone,^35^,^41^ whereas one study evaluated EtS in combination with EtG.^31^ Consistently, these biomarkers provided fair to excellent specificity—EtG (range, 71%–100%; median, 87%); EtS (range, 97%–100%; median, 98%); CDT (range, 71%–100%; median, 95%); GGT (range, 71%–100%; median, 95%); and MCV (both values 100)—but exceptionally poor sensitivity—EtG (range, 0%–97%; median, 23%); EtS (range, 7%–15%; median, 7%); CDT (range, 5%–40%; median, 13%); GGT (range, 11%–50%; median, 25%); and MCV (values of 15 and 20).

One study evaluated postnatal serum levels of insulin-like growth factor-II (IGF-II) as predictors of FASD status in children or youth who either had a history of meconium FAEE levels above 2 nmol/g or had been adopted from Eastern European countries with confirmed PAE (two point estimates).^27^ The participants were assessed for IGF-II levels below the 5th percentile. IGF-II levels below the 5th percentile had excellent specificity (99% and 100%, respectively) for predicting FASD status, but very poor sensitivity (13% and 39%, respectively) and overall accuracy (24% and 47%, respectively).

One study provided limited information on aspartate aminotransferase (AST) and alanine aminotransferase (ALT), which are both markers of impaired liver functioning, as biomarkers of PAE.^46^ Only AUC values were provided, and these were poor (0.47 and 0.54, respectively).

Phosphatidylethanol (PEth) is a more recent biomarker of ethanol metabolism that has been evaluated in maternal and infant plasma and dried blood spots.^35^,^41^,^52^–^55^ Six different studies found considerable variability in the predictive characteristics of PEth depending on the source of the PEth. Assays of maternal blood as well as plasma from the umbilical cord yielded a wide range of specificity (range, 9%–100%; median, 96%), sensitivity (range, 0%–100%; median, 22%), and overall accuracy (range, 51%–91%; median, 71%). Tests of dried blood spots taken from infants also had variability in their predictive characteristics but were generally not as good as maternal blood and plasma obtained from the umbilical cord—specificity (range, 42%–100%; median, 95%); sensitivity (range, 32%–63%; median, 52%); and overall accuracy (range, 48%–50%; median, 50%).

Collectively, these results regarding the validity of biomarkers for predicting PAE status suggest that a positive response was not very effective in identifying the full range of individuals who self-reported prenatal alcohol use and missed many affected individuals. This was also true of the studies evaluating the predictive modeling of the impact of PAE (see Appendix 2). Combining biomarkers did not result in substantial improvements in the predictive characteristics (see Figure 3A, bottom right panel). As has been observed in other biomarker analyses, there appeared to be a trade-off such that as sensitivity of combined biomarkers increased compared with single biomarker predictors, specificity was reduced.

A promising biomarker with limited predictive data reported in one study was proteins and cytokines found in the placenta.^56^ Specifically, proteins that influence angiogenesis as well as pro-inflammatory and anti-inflammatory cytokines were evaluated in a group with a history of PAE. The study only provided information on AUC, which reflects the integration of sensitivity and specificity characteristics; however, these data were in the fair to excellent range (range, 0.70–1.00; median, 0.79). In contrast to previous biomarker data, integration of different predictors resulted in improved prediction. Combined analysis of the levels of three proteins (i.e., ANX-A4, CCM-3, and VEGFR2) yielded an AUC of 1.00, and a combined analysis of another six proteins (VEGFR1, angR, VEGF-A, VEGF-C, VEGF-D, and beta-fibroblast growth factor) resulted in an AUC of 0.94. Combined cytokine levels also had good to excellent AUC values, with six pro-inflammatory cytokines (IL-1-beta, IL-2, IL-8, IL-12p70, interferon-gamma, and tumor-necrosis factor alpha) yielding an AUC value of 0.92 and four anti-inflammatory cytokines (IL-4, IL-6, IL-10, and IL-13) resulting in an AUC value of 0.83.^56^

Finally, circulating microRNAs (miRNAs) in maternal blood, which reflect epigenetic changes in response to PAE, have been explored as a potential biomarker in a sample of Ukrainian mother-infant dyads.^57^ Levels of miRNAs were compared among pregnant women without PAE; pregnant women with heavy PAE whose children were impacted; and pregnant women with heavy PAE whose children were not impacted in either growth, dysmorphology, or brain development. Heavy PAE was defined as weekly heavy episodic or binge drinking (i.e., five or more standard drinks), five or more episodes of three to four standard drinks, or 10 episodes of one to two standard drinks. Impact of PAE on the offspring was assessed by trained physicians who completed a dysmorphology assessment and by psychologists who completed a neurodevelopmental evaluation with the child. Several miRNAs (*n* = 21) were identified as differing between the exposed–affected group and both other groups, and a random forest analysis was used to predict group membership while controlling for other group differences (i.e., maternal smoking). Seven of the top 10 variables retained in the initial predictive model were miRNAs. The most common miRNAs identified were likely to influence downstream pathways related to fetal and placental growth. Specificity was excellent (91%) and sensitivity (82%) was good for miRNA levels obtained in pregnancy; however, both specificity (74%) and sensitivity (77%) were only fair for changes in the miRNA levels over the course of the pregnancy. Although this was only one study, the findings suggest that assessments of levels of specific miRNAs obtained in pregnancy may improve sensitivity in predicting PAE-related outcome compared with other biomarkers that could be obtained in pregnancy.

### Screening Tools

Screening tools were divided into five types of assessments, including facial features, neurophysiological responses in infants and older children, neuroimaging, questionnaire responses, and performance measures (see Appendix 3). In some cases, combinations of facial data and performance measures were used in predictive modeling; these are included in the performance measure section of Appendix 3.

#### Facial features

Eight studies have explored facial features as key predictors of an FASD-related diagnosis using in-person measurements and two-dimensional (2D) and three-dimensional (3D) photographs.^58^–^65^ Specificity values were variable, ranging from poor to excellent, with only a couple of studies reporting levels in the fair to poor range (range, 43%–100%; median, 86%). Sensitivity levels also were in the good to fair range (overall range, 43%–100%; median, 92%), with the exception of one study where sensitivity using the facial analysis software of 2D pictures was in the poor range. Accuracy for prediction was typically in the fair to good range (range, 79%–100%; median, 93%). Advancing technology from in-person measurement to 3D computerized configural methods did not necessarily result in improved predictive characteristics, but comparisons are complicated because samples were from different countries (i.e., United States, South Africa, Germany, and Finland), and different methods were used for defining the outcome (variations of FAS and FASD, heavy alcohol-exposed) and reporting predictive results.

More recently, one study evaluated the use of a schema that coded alterations to ocular development to differentiate individuals with a clinical diagnosis of FASD.^66^ The coding schema captured elements of visual acuity, refraction, strabismus/binocular function, and ocular structural abnormalities, with each area being coded from 1 to 4. Cut-off values of the total score (10 and 9) were evaluated relative to healthy controls; children with attention-deficit/hyperactivity disorder (ADHD); children who were born prematurely (moderate to late); and children with Silver-Russell syndrome, a genetic condition with growth impairment and neurodevelopmental compromise.^67^ Similar to attempts to capture facial features, specificity was good to excellent (88%–100%), but sensitivity was poor (43%–57%). AUC estimates were variable, ranging from 0.60 to 0.92, with the higher estimate reflecting comparisons to healthy controls.

#### Infant neurophysiology

Early identification of alcohol-related brain impairment has been attempted using indices of infant neurophysiological responses, including eye-blink conditioning^68^ and cardiac orienting response (COR).^69^,^70^ These procedures use physiological responses in the context of a learning paradigm that can be implemented with infants. For eye-blink conditioning, classical conditioning is used where an unconditioned stimulus (i.e., puff of air) that elicits a reflexive eye blink is paired with a conditioned stimulus (i.e., auditory tone or picture) over repeated trials. After many pairings, the conditioned stimulus is then able to elicit the eye-blink response. Rate of learning is assessed by the percentage of pairing trials of the conditioned stimulus with the unconditioned stimulus needed before the eye blink is elicited by the conditioned stimulus in the absence of the unconditioned stimulus. In the case of COR, heart rate responses are monitored while stimuli (i.e., auditory tone or picture) are presented over several trials, referred to as habituation trials, and then after presenting novel but similar stimuli over several trials (dishabituation trials). Heart rate typically decelerates in response to novel information and returns to baseline over the course of several habituation trials; it decelerates again in response to the second novel stimulus. The magnitude of the deceleration in the first three habituation trials is believed to reflect the infant’s encoding of stimuli, whereas the magnitude of the first three dishabituation trials reflects the infant’s ability to differentiate the first and second related stimuli, indexing early memory functioning. These methods are advantageous as standardized early assessments of cognitive functioning often are not adequate in assessing alcohol-related brain impairment.

Eye-blink conditioning was reported in one study that provided data for its predictive utility relative to FAS and to a broader spectrum of individuals with heavy PAE, defined as averaging at least 1.0 oz absolute alcohol per day or ≥ five standard drinks per occasion in the first trimester of pregnancy; and a group defined as having FASD.^68^ Eye-blink conditioning had a sensitivity of 100% for FAS prediction, but this fell to 70% for prediction of a broader spectrum of heavy PAE and FASD. Specificity was comparable for both predictive models at 75%. Overall accuracy was 82% for predicting FAS and 72% for predicting heavy PAE/FASD. The PPV value was 87% for heavy PAE/FASD and 63% for FAS alone, and NPV was 51% for predicting heavy PAE/FASD and 100% for FAS alone.

Findings for COR were not reported in terms of sensitivity, specificity, and overall accuracy but were reported in terms of PPV, NPV, and AUC values in two different articles using overlapping samples of Ukrainian mother-infant dyads.^69^,^70^ Using the key features of COR (i.e., speed of the response, average trough), a PPV of 82%, an NPV of 62%, and an AUC value of 0.81 were reported in one of the studies for predicting neurodevelopmental impairment at 12 months.^70^ Only small incremental gains were obtained when including maternal drinking information in the model. In the second study, an index score derived from the visual COR data had an AUC value of 0.77 for predicting later preschool FASD status.^69^ These results suggest that early neurophysiological responses may be useful in improving identification of individuals with neurodevelopmental impairment in infancy, which has often been a key factor limiting early diagnosis.

#### Neurophysiology with older children

Neurophysiological responses assessed in older children have included auditory evoked potentials and eye-tracking or saccadic eye movements. One study evaluated auditory evoked potentials, which assess the time it takes for a signal to travel along the auditory nerve track in response to sound stimuli.^71^ Auditory evoked potentials by themselves had fair sensitivity (79%) and poor specificity (43%) and overall accuracy (61%). However, when various indices of P300 responses were combined (e.g., latency, magnitude), increased differentiation of individuals with FASD from individuals with Down syndrome was found (sensitivity, 79%; specificity, 86%; and overall accuracy, 82%).

Eye-tracking movements also have been used to identify children impacted by PAE.^72^ Two studies provided data regarding predictive validity of eye-tracking measures in individuals with FASD.^73^,^74^ Accuracy ratings ranged from poor (65%) to excellent (90%). Combining eye-tracking information with data obtained from diffusion tensor imaging and neurobehavioral testing resulted in improved accuracy in one study (range of 65%–76% improved to 85%).^73^ Eye-tracking movements also have been used to predict the impact of other neurodevelopmental disorders,^75^,^76^ suggesting the importance of studies that attempt to establish differential predictive validity for the effects of PAE relative to other neurodevelopmental disorders (e.g., autism). This likely is also true of the infant neurophysiological measures (i.e., COR and eye-blink conditioning), which also have been used to determine mean group differences between other clinical groups and typically developing controls.^77^,^78^

#### Neuroimaging

Three neuroimaging studies provided predictive data for the impact of PAE.^73^,^79^,^80^ Using weighted volumetric scores of specific brain regions, specificity was good (88%), but sensitivity was still in the poor range (64%).^80^ The combination of four key features of diffusion tensor imaging also provided relatively poor accuracy (67%) in predicting an FASD diagnosis.^73^ Excellent specificity (95%) was reported for measurement of the “hook” area of the corpus callosum, but sensitivity of this measurement was poor (52%), suggesting that this method did not identify those impacted by PAE at better than chance levels.^79^ This suggests that, like other biomarker prediction of PAE and PAE impact, prediction based on neuroimaging findings provides a clear signal of PAE or its impact, but is not sufficiently sensitive to capture the range of impact commonly seen in individuals exposed to alcohol.

#### Parent questionnaire measures

Six identified studies reported predictive characteristics of caregiver or provider responses to a questionnaire in identifying children with alcohol exposure or FASD.^81^–^86^ Parental responses to questionnaires developed specifically for identifying children impacted by PAE or standardized measures used to flag aspects of alcohol teratogenesis typically had good to excellent specificity (overall range, 66%–96%; median, 83%); only one study using subsets of items from the Child Behavior Checklist yielded sensitivity in the poor to fair range.^82^ Sensitivity reported in these studies was poor to excellent (range, 54%–100%, median, 85%), with the lowest sensitivity reported in a study attempting to differentiate only pFAS in one analysis (54%).^85^ Relatively few studies reported overall accuracy rates, which ranged from poor to excellent (range, 68%–94%; median, 71%). The wide range in predictive characteristics of these types of data was dependent on the definition of the predictor (PAE, pFAS, FAS, or FASD) and the comparison group used—typical healthy controls or controls with ADHD. Incomplete evaluation of those who screened negative also may have overinflated estimates in one study of the predictive characteristics as this method fails to include the possibility of false negatives in the screening process.^84^

#### Child performance measures

Nine studies identified predictive characteristics of child performance measures and combinations of performance measures and other indicators of PAE or FASD.^87^–^95^ These ranged from quick screening tests to complex neurobehavioral batteries in isolation or in combination with dysmorphology information. Of these nine studies, one assessed the predictive characteristics of motor assessments,^92^ whereas another two studies looked at aspects of narrative speech only.^94^–^95^ Specificity ratings for all nine studies ranged from poor (45%) to excellent (100%), and sensitivity ratings ranged from poor (2%) to excellent (100%). Overall accuracy in these studies also ranged from poor (49%) to excellent (100%). Two of the nine studies compared individuals with PAE to both typical healthy control groups and to other clinical groups separately or in combination with the healthy control group.^89^,^90^

## Discussion and Conclusions

Identifying children who have been prenatally exposed to alcohol or, more importantly, have been negatively impacted by their exposure continues to be an important area of investigation. Although a range of biomarkers and screening tools have been explored, there is no agreed-upon procedure or method that provides excellent sensitivity, specificity, and overall accuracy, suggesting the need for continued research. A general theme found in the existing literature is higher specificity then sensitivity for many of the biomarkers and screening tools used to identify PAE and its impact. This means that although researchers and clinicians often have confidence when they identify PAE or its impact, they struggle with capturing the full range of individuals impacted. Exceptions to this were found in recent investigations of biomarkers of PAE using miRNAs related to growth and vascular development,^57^ proteomic changes associated with PAE,^56^ and combinations of markers estimating levels of various cytokines.^56^ However, replications of these findings across other samples are needed to confirm the limited data currently available on the predictive characteristics of these biomarkers.

For predicting the outcomes of alcohol teratogenesis, facial features operationalized using varying methods (i.e., in person, 2D, or 3D) provided relatively high sensitivity, specificity, and accuracy, but a few point estimates were less effective. Neurophysiological responses assessed in infancy and later childhood were able to differentiate individuals impacted by PAE, but the upper limits of prediction were in the fair to good range. Moreover, there was some indication that these responses were better at defining pFAS/FAS rather than the full spectrum of FASD, including heavy PAE. Neuroimaging methods, including volumetric and diffusion tensor imaging, also had high specificity but poor sensitivity, similar to biomarkers of PAE alone. Parent and professional responses to questionnaires had both good sensitivity and specificity, with the exception of one comparison that attempted to discriminate specific subgroups of FASD. This increased sensitivity relative to other biomarkers and screening tools may be biased by the fact that all studies in this area involved clinical FASD samples, which may reflect shared variance associated with the parent seeking treatment for the child. Replications in prospective cohorts of exposure may be helpful in clarifying this potential bias in predictive validity. Child performance measures had varying ranges of success in predicting those impacted by PAE, which seemed to vary as a function of inclusion of other biomarkers and the nature of the comparison sample utilized in the prediction.

### Limitations in the Existing Literature

The definition of the criterion to be predicted was problematic across studies. Maternal report of PAE or heavy PAE was operationalized using multiple different methods that were integrated in different ways (e.g., summed, any positive response, principal component analysis of several responses). Moreover, results appeared to vary as function of the context in which the maternal self-report was collected. In one study, maternal self-report of PAE was higher than PAE confirmed using biomarker data.^52^ In another study in the context of a health care environment, however, estimates of PAE using these methods were in the opposite direction.^96^ Even in studies of FAEE levels that were conducted in the same hospital setting where participants were assured of confidentiality, FAEE levels were dramatically higher when they were sampled from de-identified meconium, which did not require maternal consent, than when informed consent from the mother was needed.^97^ Mothers with the heaviest prenatal alcohol use were more likely to self-select out of the study,^98^ most likely in response to the stigma associated with PAE.^99^

A number of studies used other biomarkers to validate a novel biomarker. Convergent validity is useful in verifying the validity of the novel biomarker but limits the window of detection between biomarkers; moreover, threshold or cutoff values used to signal a positive test also varied. Often biomarkers reflect severe alcohol use disorder as they are indicators of damage to organs (e.g., liver) over a prolonged period; however, these methods often failed to capture the full range of FASD or PAE that can have adverse impact on a developing fetus. Other biomarkers are byproducts of the metabolism of alcohol and have limited windows for detecting PAE. For each biomarker, other factors also may reduce the validity of their prediction, including personal care and hygiene (e.g., corruption from chemicals used in hair and nail care), other foods that may produce alcohol metabolites during decomposition,^97^ and willingness of the mother to provide the biological sample. Some investigators have opted to use a combined approach, although costly, to predict PAE status^35^,^45^,^53^ to compensate for the individual weaknesses or limitations of any one method of identification of PAE.

Many studies used an FAS or FASD diagnosis as the outcome, but diagnostic formulations used in the field vary considerably, and evidence suggests that the degree of agreement across methods is low.^12^ The development of a consensus diagnostic formulation for individuals with FASD would be helpful in reducing error variance associated with the diagnostic formulations. As mentioned previously related to parental questionnaires as screening tools, use of clinical samples also is biased because it selects for individuals who sought care for the treatment of the child. This can result in circularity in defining the screening tool as the predictor when the screening tool may be drawn from the same construct domain or type of test used to categorize or diagnose the clinical group. Implementation of screening approaches across multiple samples—including both clinical and prospective cohorts of PAE from diverse populations that vary in ethnic, geographic, and cultural backgrounds—may help with eliminating these biases.

Another limitation of some studies was that they provided predictive estimates but failed to sample the criterion within the entire pool of individuals screened.^59^,^84^ This approach occurred in larger screening cohorts where individuals who screened negative were not sampled further and were assumed to be true negatives. These assumptions may result in overestimation of the predictive characteristics of the biomarker or screening tool.

Sensitivity and specificity characteristics are independent of the prevalence of the condition under investigation (e.g., PAE), but accuracy, PPV, and NPV are influenced by the rate of PAE or individuals impacted by PAE in a study’s sample (see Figure 1 for computational formulas). Considerable variation existed across studies in the ratios of affected and nonaffected individuals in the sample. In many studies, both groups were comparable in size, which results in an estimate of the predictive characteristics under circumstances where the prevalence of the condition in the sample is substantially higher than the rate anticipated in the general population. Changes in the sensitivity and specificity of a biomarker if the prevalence of the condition deviates from 50% can result in reduced validity of estimates of the overall accuracy of a biomarker or screening tool.^100^ This suggests that the accuracy ratings commonly found for biomarkers of PAE and its impact may be overweighted by their high specificity and that these biomarkers are less predictive in real-world settings where the prevalence has been estimated to fall between 5 to 50 per 1,000 children.^18^,^19^ Implementation of biomarkers or screening tools in clinical trials in the context in which they are intended to be used may help to evaluate the true accuracy of these tools.

The studies surveyed also differed in comparison samples used, with some studies including typical healthy controls and others attempting to differentiate offspring with PAE relative to other clinical groups who might present for diagnosis. Estimates of predictive validity of biomarkers or screening tools relative to typical healthy controls are often higher than those found when using a clinical comparison group. However, the latter approach provides a better estimate of the usefulness of a biomarker or screening tool to clinicians asked to determine if a given child has been impacted by their PAE. In evaluating biomarkers or screening tools, researchers should consider a tiered approach with a first evaluation relative to typical controls, followed by evaluation relative to other clinical groups to improve understanding of the clinical utility of the biomarker or screening tool. The final tier would then involve an actual clinical trial of the clinical utility of the biomarker or screening tool and an assessment of where it fits within a clinical diagnostic algorithm—that is, whether it functions more as a screener that can flag the need for other diagnostic assessments or as an actual diagnostic tool, indicating its high concordance with the clinical endpoint.

Finally, the scalability of a biomarker or screening tool is also important to consider. The financial cost of the assay or test and the expertise needed to carry out an assessment can dramatically limit the utility of a given biomarker or screening tool, particularly in countries with low resources. The gold standard for diagnosis is a multidisciplinary team assessment that includes at a minimum a physician who can assess alcohol-related dysmorphology and a psychologist who can assess neurobehavioral impairment. Even if variations in diagnostic criteria utilized among existing clinics are resolved, this method of identification in no way can meet the needs of those impacted by PAE given the recent prevalence estimates. This is true in countries with considerable resources as well as in those with minimal resources. Therefore, when designing biomarkers or screening measures, it is important to consider to what extent the test can be implemented globally with limited expense and expertise.

### Limitations of This Review

This review was not intended to be a comprehensive review of each biomarker as several studies were eliminated that characterized biomarkers in different populations, established group differences, or estimated costs associated with implementation. Several existing reviews have provided in-depth discussions of one or more biomarkers or screening tools with greater details on the ease of collection, detection windows, limits of detection, costs, and feasibility of use.^101^–^104^ This article aimed to focus on the predictive characteristics of biomarkers and screening tools to assess PAE and its associated impact. The search process using the selected terms may have missed relevant articles as several additional papers were found among the references in those articles identified using the initial search terms. Also, most biomarkers did not have sufficient numbers of studies for a true meta-analysis given the variation in threshold or cutoff values used to define risk and in the predictor. As a result, the range and median value of data obtained from the articles were provided. Providing uniform data-reporting formats in future studies would help with subsequent attempts to integrate these types of studies.

### Future Directions

The relative importance of the predictive validity characteristics depends on the goals of the screening and on the diagnostic algorithm in which the biomarker or screening tool is being used. PPV and NPV only incorporate validity of a positive or negative test signal, respectively, and are most useful for clinicians trying to interpret a biomarker or screening tool result relative to a clinical endpoint. Accuracy provides a summary of the overall correctness of the biomarker or screening tool, but does not fully capture its errors (i.e., false positives and missed cases). In cases where the costs of these errors are high, accuracy is an inadequate indicator of success. One could argue that this is the case for PAE and its associated impact, where false positives could potentially be stigmatizing and missed cases would limit opportunities for harm reduction and intervention during early periods of neuroplasticity. Many biomarkers and screening tools related to PAE have good specificity, but their implementation requires further evaluation of the cost-benefit ratios of use within given environments and discussions regarding the ethics of implementation relative to patient privacy and autonomy. Much progress is needed in the development of biomarkers and screening tools to improve sensitivity, which is likely to be most valued by individuals affected by PAE and those who care for them as low sensitivity results in lost opportunities for harm reduction and early intervention. AUC values provide a tool for estimating predictions that capture both sensitivity and specificity elements but may obscure relative weaknesses in one or the other. Ultimately, final decisions on clinical implementation should include input from key stakeholders who may assign different value judgments to these predictive characteristics.

Improvements in the predictive characteristics of biomarkers and screening tools would have important ramifications for surveillance methods and clinical care of individuals negatively impacted by PAE. Surveillance methods that use biomarkers or screening tools currently are limited by the low sensitivity of most available biomarkers and screening tools because a negative test result does not exclude individuals who may be negatively impacted by PAE. Surveillance studies that assume those who screen negative are unaffected and do not conduct further evaluations therefore may be underestimating true case prevalence rates. The clinical use of biomarker or screening tools also has been limited by insufficient data on predictive utility characteristics in published studies. Moreover, implementation within clinical environments often only takes place if researchers are exploring the use of the biomarker or screening tool in their studies. Improved reporting of the predictive validity characteristics of these measures are needed before consensus could be reached to support larger-scale implementation of these biomarkers and screening tools.

The field of alcohol teratogenesis initially sought to determine if PAE resulted in group differences from offspring not exposed to PAE on a variety of outcomes; however, future efforts also need to include efforts to help identify affected individuals. Predictive validity information moves beyond mean group differences and attempts to determine if a given measure’s dispersion is such that a threshold, cutoff value, or rule based on an outcome or a cluster of outcomes could be used to identify those impacted by PAE. In most cases, these differing aims could be achieved within the same study, using different analyses to help with identifying better biomarkers that can improve early identification and access to habilitative care.

There are many promising areas where group differences have been explored but predictive characteristics have not yet been reported. One promising diagnostic tool may involve functional near-infrared spectroscopy,^105^,^106^ which assesses changes in oxygenation levels of brain tissue by shining near-infrared light through the scalp that is then differentially reflected back to a sensor as different light wavelengths depending on whether or not the blood is oxygenated. Individuals with FASD show specific patterns of buildup of deoxygenated hemoglobin over time in response to prefrontal cortex activation that differ both from typically developing children and from those with other neurobehavioral impairments. Epigenetic changes, including DNA methylation, histone modifications, and other miRNAs associated with PAE, may also be effective biomarkers,^107^,^108^ although diagnostic analyses of these measures have rarely been reported. One promising study assessing changes in DNA methylation (i.e., the process of adding methyl groups to a DNA molecule) found that children with PAE and their mothers both had higher DNA methylation levels of proopiomelanocortin and PER2, a gene involved in regulating circadian rhythms, resulting in reduced expression of these genes. In contrast, postnatal choline supplementation, which increases the bioavailability of additional methyl groups after birth, resulted in reduced DNA methylation and increased expression levels of these stress-regulatory genes.^109^ In addition, the health consequences of PAE are just beginning to be explored, and it may be important to determine to what extent these consequences may help identify individuals impacted by PAE.

Going beyond group differences to establish the diagnostic test validity of an outcome relative to healthy children without PAE and then relative to other children with other neurobehavioral conditions will provide the needed information to evaluate effectively whether these potential biomarkers will have clinical utility and should be further evaluated in the context of a biomarker clinical trial. This transition to a systematic process of biomarker and screening tool evaluation is needed to address the public health need of improving early identification of individuals impacted by PAE so that harm reduction and habilitative care efforts can be implemented.

## Figures and Tables

**Appendix 1:** Predictive Characteristics* of Biomarkers for Prenatal Alcohol Exposure Status

Author	Subject Description	PAE/FASD Sample Description	Control Sample Description	Biomarker	Source of Biomarker	Outcome	Sensitivity	Specificity	PPV	NPV	Accuracy	AUC From ROC

Azurmendi-Funes et al., 2019^46^	Mothers at a public maternity hospital in Spain	20 High risk due to PAE (20 g alcohol or ≥ 3 binges of ≥ 40 g alcohol)	71 Non–high-risk PAE	%CDT (cutoff ≥ 95%)	Maternal blood	Self-report of PAE	25.0	93.0		81.5		.75/.71
				AST								.47
				ALT								.54
				GGT								.42
				MCV								.38

Bakdash et al., 2010^38^	Meconium samples	116 FAEE (≥ 100 ng/g)	480 FAEE (< 100 ng/g)	EtG ≥ 500 ng/g	Meconium	FAEE > 100 ng/g	78.6	98.7	82.5	98.4	97.3	

Bakhireva et al., 2014^35^	Women from a specialty treatment clinic	28 PAE	32 No PAE	PEth > 8 ng/mL delivery	Maternal blood	Self-report of PAE	22.2	100				
				GGT > 40 U/L mid-gestation			14.8	100				
				GGT > 40 U/L delivery			10.7	86.2				
				%CDT > 2.0% mid-gestation			3.7	100				
				%CDT > 2.0% delivery			10.7	96.7				
				EtG ≥ 25 ng/mL mid-gestation	Maternal urine		14.8	96.9				
				EtG ≥ 25 ng/mL delivery			7.4	96.9				
				EtS ≥ 7 mid-gestation			14.8	100				
				EtS ≥ 7 delivery			7.4	96.9				
				PEth (> 8 ng/mL)	Infant blood spots		32.1	100				
				FAEE (sum of 9)	Infant meconium		28.6	81.3				
				All mid-gestation			32.1	96.9				
				All delivery + infant PEth (mid-gestation/both)			50/50	81.3/93.8				

Bearer et al., 1999^34^	Mothers and infants from a large urban hospital enrolled in a study on the effects of cocaine	127 Positive FAEE	92 Negative FAEE	FAEE sample peak at 0.02 minutes/standard for ethyl linoleate	Meconium	Periconceptual self-report of PAE (variation in dosage level of self-report)	64–81	43–48				
						Trimesters 2 and 3 self-report of PAE (variation in dosage level of self-report)	66–72	66–72				

Bearer et al., 2003^36^	Mothers and infants from South Africa	27 Meconium samples with varying levels of ethyl linoleate	None	FAEE ethyl oleate ≥ 32 ng/g	Meconium	Self-report of PAE (≥ 1.5 AA/drinking days)	84	83	94	63		.92
				FAEE ethyl oleate ≥ 13 ng/mg			100	66.7	91	100		
				FAEE ethyl oleate ≥ 77 ng/mg			68.4	100	100	50		

Bearer et al., 2005^28^	Postpartum women in large urban teaching hospital in Cleveland, Ohio (*n* = 248), and Muslim women receiving care at a hospital in Jordan (*n* = 30)	169 Nonabstainers	55 Abstainers/ nondrinkers (30 Jordan and 25 Cleveland)	FAEE score derived from principal component analysis of variance	Meconium	Self-report > 7 drinks per drinking day	78–88	59–63	7–18	98–99		.69–.75
						Maternal self-report > 21 drinks/drinking day	72–84	50–60	7–15	95–98		.60–.71

Cabarcos et al., 2014^39^	Infant meconium samples	FAEE ≥ 600 ng/mg; ethyl myristate, ethyl palmitate, ethyl stearate, and EtG	FAEE < 600 ng/mg; ethyl myristate, ethyl palmitate, ethyl stearate, and EtG	FAEE ≥ 600 ng/mg; ethyl myristate, ethyl palmitate, ethyl stearate, and EtG	Meconium	EtG (> 50 ng/g)					61.7	

Chan et al., 2004^99^	Mothers from Toronto, Canada (*n* = 104) and Israel (*n* = 103)	6 Heavy PAE and 73 with FAEE above lower limit of detection	None	Women with AUD or FAEE ≥ 2 nmol/g	Meconium	Self-report and clinical referral of AUD	100	98	63	100		

Eichler et al., 2016^47^	Meconium samples from infants whose mothers were seen in pregnancy and when child was 6–8 y	43 Self-reported PAE in trimester 3	137 Self-reported no PAE	EtG > 10 ng/g	Meconium	Self-report of PAE in trimester 3	33.3	79.0	32.6	79.6	68.3	
				EtG > 120 ng/g	Meconium	Self-report of PAE trimester 3	18.6	86.9	30.8	77.3	70.6	

Ferraguti et al., 2017^48^	Women who attended a gynecology and obstetrics hospital in Rome	46 Alcohol drinkers	24 Abstainers	EtG > 500 ng/mL	Maternal urine	Self-report of PAE	40.9	75.0	60.0	58.1	58.7	

Gauthier et al., 2015^29^	Mothers of infants admitted to the newborn intensive care unit in a large inner-city hospital	11 “Drinkers” as defined by a positive AUDIT item	56 Nondrinkers	FAEE ethyl stearate	Placental FAEE	Maternal AUDIT+	82.0	87.0	50.0	97.0		.93
				FAEE ethyl linoleate			82.0	83.0	44.0	97.0		.88
				FAEE OLL			82.0	94.0	70.0	97.0		.91
				FAEE OLL + stearate			82.0	93.0	64.0	97.0		.92

Goecke et al., 2014^30^	Women attending outpatient visit at obstetrics and gynecology department at the University of Erlangen-Nuremberg, Germany, FRAMES	204 Self-reported PAE	782 Self-reported no PAE	Ethyl stearate > 0 ng/g	Meconium	Self-report of PAE	32.5	76.7	27.3	80.8	67.3	
				EtG ≥ 0 ng/g			26.96	85.7	33.7	81.4	73.3	

Gomez-Roig et al., 2018^49^	Pregnant women seen at a maternal fetal and neonatal medicine hospital	99 Gestational alcohol users	54 Abstinent women	EtG ≥ 7.0 pg/mg	Maternal hair	Self-report of PAE	2.0	96.3	50.0	37.0	37.3	

Gutierrez et al., 2015^41^	Biomarkers in Pregnancy Study Women attending UNM prenatal clinic providing care to women with SUD and addiction	42 PAE	43 No PAE	EtG ≥ 8 pg/mg	Maternal hair	Self-report of PAE (composite index)	19.1	86.1	57.1	52.1		
				GGT > 40 U/L			20	97.6	88.9	56.1		
				%dCDT > 2.0%			5	100	100	52.5		
				%dCDT > 1.7%			22.5	71.4	42.8	49.2		
				uEtG ≥ 25 ng/mL			4.9	97.6	66.7	51.2		
				EtS ≥ 7 ng/mL			7.3	97.6	75.0	51.9		
				PEth ≥ 8 ng/mL			17.5	100	100	56.6		

Himes et al., 2015^31^	Meconium samples from Safe Passages Study of the Prenatal Alcohol in SIDS and Stillbirth Network	58 Women who drank past 19 weeks in pregnancy	33 No PAE	Maternal self-report of PAE	Meconium	FAEE sums (4–9)	52–64.9	45.1–51.4				
						EtG (10–50 ng/g)	68.6–82.4	71.4–75.0				
						EtG (333 or 444 ng/g)	96.7–96.8	71.4–75.0				
						EtG or EtS (> 2.5 ng/g)	66.7–83.3	64.6–76.3				
						FAEE or EtG/EtS	70.4–75.0	61.7–78.4				

Holbrook et al., 2019^56^	UNM Clinic ENRICH Cohort (prenatal clinic for individuals with SUD)	13 PAE as defined by AUDIT Score ≥ 8 total consumption of ≥ 84 drinks, positive ethanol biomarker	13 No PAE	Angiogenesis-related proteins Western blot		PAE group via multiple determination						.78–.89
				Angiogenesis-related proteins Meso scale								.76–.81
				Pro-inflammatory cytokines								.70–.78
				Anti-inflammatory cytokines Meso scale								.70–.76
				ANX-A4, CCM-3, VEGFR2,								.100
				VEGFR1, angR, VEGF-A, VEGF-C, VEGF-D, beta-FGF								.94
				6 Pro-inflammatory cytokines								.92
				4 Anti-inflammatory cytokines Meso Scale								.83

Howlett et al., 2018^42^	Pregnant women from England seeking prenatal care in first trimester; Biosample study with no consent	20 Positive for GGT	567 Negative for GGT	CDT ≥ 1.60% and ≥ 1.87 for probable chronic alcohol use	Maternal blood	GGT ≥ 45 U/L	5.00	98.8	12.5	96.7	95.6	

Joya et al., 2016^50^	Mother-infant dyads from Spain	19 Self-reported some alcohol consumption in pregnancy	61 Self-reported no alcohol consumption	EtG (> 11 pg/mg)	Maternal hair	EtG meconium (≥ 30 ng/g vs. < 30 ng/g)	76.2–85.7	73.7–78.9				

Kwak et al., 2014^26^	Mothers from Korea who reported PAE	8 Heavy drinkers; 19 moderate, 85 light; FAEE for 54 for light/moderate	182 No PAE	FAEE ≥ 20 nmol/g	Meconium	Self-report of PAE	3.7	98.9	50.0	77.6	77.1	.52
				FAEE ≥ 2 nmol/g			22	73.6	20	76.1	61.7	
				FAEE ≥ 10 nmol/g			5.6	94.5	23.1	77.1	74.2	

Kwak et al., 2014^55^	Mothers from Korea who reported PAE	8 Heavy drinkers; 22 moderate, 87 light; contrasted moderate to heavy (*n* = 30)	188 No PAE Light drinkers = 87	PEth 4.2 nmol/L	Maternal blood	Maternal self-report of PAE	100	96.4				.99
				PEth 3.8 nmol/L			66.7	96.4				.85
				PEth 1.2 nmol/L			40.7	95.4				.69

Lamy et al., 2017^51^	Meconium samples collected from Rouen (Normandy)	20 Self-reported PAE	607 Self-reported no PAE	EtG > 60 ng/g	Meconium	Self-report PAE in trimester 3	5	97.4	5.9	96.9	94.4	

Maxwell et al., 2019^53^	Neonates born at a tertiary care center in Charleston, West Virginia	43 PEth positive	119 PEth negative	PEth DBS ≥ 8 ng/ml	Umbilical Cord	Self-report of PAE	4.7	98.3	50.0	74.1	73.5	
						Urine PEth	0.0	97.4	0.0	72.6	91.2	
						Mom peripheral blood PEth	3.9	9.0	12.5	74.0	69.2	

May et al., 201852	Mothers from Western Cape province in South Africa	126 Self-reported PAE	67 Self-reported no PAE	PEth > 8 ng/g	Maternal DBS PEth/Fingernail clipping (EtG)	Self-report prior 7 days	92.0	58.7	50.0		68.9	
				PEth > 8 ng/g		Self-report prior 21 days	72.1	83.0	91.2		75.1	
				EtG > 8 ng/g		Self-report prior 7 days	65.0	72.9	52.0		70.5	
				EtG > 8 ng/g		Self-report prior 21 days	50.7	92.5	94.7		62.2	
				One or both		Self-report prior 7 days	95.0	48.9	45.6		69.2	
				One or both		Self-report prior 21 days	80.0	75.5	89.6		78.8	

Ostrea et al., 2006^33^	Mother/infant dyads	93 Self-reported PAE	31 Self-reported no PAE	Ethyl linoleate > .05 μg/g	Meconium	Self-report any alcohol	26.9	96.8	96.2			.60
				Ethyl arachidonate > .20 μg/g			18.3	96.8	94.4			.57

Sarkola et al., 2000^45^	Women with a history of SUD who were attending a special outpatient clinic	44 used alcohol and drugs; 8 of 13 who drank heavily had child with FAE impact; 31 drank moderately	62 Controls	GGT	Maternal blood	Group status (heavy vs. moderate PAE)	30.8	75.6	40.0	71.0	63.4	.65
				MCV			15.4	100	100	73.8	75.0	.72
				CDT			7.7	93.6	33.3	70.7	68.2	.48
				CDT/transferrin			15.4	87.1	33.3	71.1	65.9	.54
				MCV and GGT			38.5	78.6	45.5	73.3	65.9	
				GGT	Maternal blood	FAE vs. other exposed (exposure and growth HC)	50.0	81.8	40.0	87.1	75.6	.76
				MCV			20.0	100	100	81.8	82.6	.87
				CDT			12.5	94.4	33.3	82.9	79.6	.60
				CDT/transferrin			25.0	88.9	33.3	84.2	77.2	.62
				MCV and GGT			62.5	81.8	45.5	90.0	78.1	

Stevens et al., 2020^54^	Pregnant women approached at antenatal clinic visits in Auckland, New Zealand	39 PEth exposure and 30 moderate to heavy exposure	26 No PEth	PEth > 8 ng/g	Infant DBS	Self-report No/Low vs. Heavy	63.0	42.1	43.6	61.5	50.8	
				PEth > 20 ng/g		Self-report No/Low vs. Heavy	52.4	45.7	36.7	61.5	48.2	
				PEth > 8 ng/g		TWEAK+	43.2	60.0	61.5	41.7	50.0	
				PEth > 20 ng/g		TWEAK+	54.6	46.9	41.6	60.0	50.0	

Wurst et al., 2008^40^	Mothers seeking routine ultrasound at university hospital	3 FAEE (≥ 2.3 pg/mg)	100 FAEE (< 2.3 pg/mg)	Hair EtG social drinking (≥ 7 pg/mg to ≥ 25 pg/mg)	Maternal hair	FAEE ≥ 2.3 pg/mg	0	87.5	0	96.6	84.9	
				Hair EtG heavy drinking (≥ 25 pg/mg)			0	95.5	0	96.6	92.3	

* Prediction characteristics evaluated in each study included sensitivity, specificity, NPV, PPV, accuracy, and AUC derived from ROC curves. Sensitivity refers to the probability that the test is positive when the condition is present. Specificity refers to the probability that the test is negative when the condition is not present. PPV refers to the probability that the condition is present when the test is positive. NPV refers to the probability that the condition is not present when the test is negative. Accuracy refers to the overall probability that the case is correctly classified from the test. Finally, AUC is derived from creating receiver operating curves by plotting the true positive rate (sensitivity) relative to the false positive rate (1-specificity). The area under the curve references the area on the graph created by the regression line relative to the chance rate of prediction. Values of 1 would indicate perfect condition, and values of 0.50 would indicate chance prediction using a yes/no model. Predictive validity values are presented as percentages with the exception of AUC values, which are reported in proportions of accurate diagnostic classification with values of 0 to 1.00.

*Note:* AA, absolute alcohol; ALT, alanine aminotransferase; angR, angR protein; ANX-A4, annexin-A4; AST, aspartate aminotransferase; AUC, area under the curve; AUD, alcohol use disorder; AUDIT, Alcohol Use Disorders Identification Test; beta-FGF, beta-fibroblast growth factor; CCM-3, cerebral cavernous malformation 3; CDT, carbohydrate-deficient transferrin; CDT/transferrin, carbohydrate-deficient transferrin to total transferrin ratio; DBS, dried blood spots; dCDT, disialo-carbohydrate-deficient transferrin; ENRICH, Ethanol, Neurodevelopment, Infant and Child Health; EtG, ethyl glucuronide; EtS, ethyl sulfate; FAE, fetal alcohol effect; FAEE, fatty acid ethyl ester; FASD, fetal alcohol syndrome disorders; FRAMES, Franconian Maternal Health Evaluation Studies; g, gram; GGT, gamma-glutamyltransferase; HC, head circumference; MCV, mean corpuscular volume; ng/g, nanograms per gram; ng/mg, nanograms per milligrams; ng/mL, nanograms per milliliter; nmol/g, nanomoles per gram; nmol/L, nanomoles per liter; NPV, negative predictive value; OLL, oleate + linoleate + linolenate; PAE, prenatal alcohol exposure; PEth, phosphatidylethanol; pg/mg, picograms per milligram; PPV, positive predictive value; ROC, receiver operating characteristic; SIDS, sudden infant death syndrome; SUD, substance use disorder; TWEAK+, TNF [tumor necrosis factor]-like weak inducer of apoptosis; uEtG, urinary ethyl glucuronide; μg/g, micrograms per gram. U/L, units per liter; UNM, University of New Mexico; VEGF, vascular endothelial growth factor; VEGFR, vascular endothelial growth factor receptor.

**Appendix 2:** Predictive Characteristics* of Biomarkers for Fetal Alcohol Spectrum Disorders and Associated Symptoms

Author	Sample Description	PAE/FASD	Control Sample	Biomarker	Source of Biomarker	Outcome	Sensitivity	Specificity	PPV	NPV	Accuracy	AUC from ROC

Andreu et al., 2019^27^	55 native Spanish children (ages 8–12) from the Meconium Project and 98 children adopted from Eastern European communities	55 native Spanish children with FASD; 33 FAEE > 2 nmol/g; of 98 children from Eastern Europe: 31 with FASD, 42 partial FAS, 6 ARBD, and 5 ARND	31 FAEE < 2 nmol/g	Serum IGF-II (below 5th percentile)	Infant blood	FASD diagnosis	12.7	100	100	14.8	24.2	
				Serum IGF-II (below 5th percentile)	Infant blood	FASD diagnosis	39.0	96.8	98.8	19.4	46.6	

Balaraman et al., 2016^57^	Ukrainian pregnant mothers at prenatal care visits	22 Heavy alcohol-affected	23 No PAE	Selected mRNAs mid to late pregnancy	Maternal blood	Defined by self-report and child outcome	81.8	91.3	90.0	84.0	86.7	
				Change in mRNAs			77.3	73.9	73.9	77.3	75.6	

Lee et al., 2018^32^	Meconium samples from infants born in hospitals— Uijeongbu St. Mary’s Hospital, Catholic University of Korea	5 with HC and length ≤ 10th percentile	FAEE < 0.5 nmol/g	FAEE 0.5 nmol/g	Meconium	Growth delay—HC, length < 10th percentile	40.0	92.8			68.0	
			FAEE < 2 nmol/g	FAEE 2 nmol/g	Meconium		20.0	98.0			68.0	
			FAEE < 0.5 nmol/g	FAEE 0.5 nmol/g	Meconium		50.0	92.8			79.0	
			FAEE < 2 nmol/g	FAEE 2 nmol/g	Meconium		25.0	98.0			79.0	

Niemelä et al., 2016^43^	All live births in Finland from 1987 to 2005 were matched to a prenatal serum specimen biobank and compared to FAS in Finnish Register of Congenital Malformations	385 Mothers of children with FAS + 95 PAE but no FAS	745 Controls (mothers no PAE and no child effects)	CDT ≥ 1.79%	Maternal blood	Self-report/ FAS diagnosis	39.5/4.2	96.4	3.6			.78/.53
				GGT ≥ 40 U/L			41.0/13.7	95	5			.82/.72
				Combo CDT–GGT ≥ 3.35			54.0/14.7	95.3	4.7			.87/.73
				EtG ≥ 0.1 mg/L			16.9/6.1	100	0			.58/.56

Niemelä et al., 2016^44^	All live births in Finland from 1987 to 2005 were matched to a prenatal serum specimen biobank and compared to FAS in Finnish Register of Congenital Malformations	385 Mothers of children with FAS	745 Controls (mothers no PAE and no child effects)	CDT ≥ 1.79%	Maternal blood	FAS diagnosis	12.5	99.3	90.6	68.7	69.7	
				GGT ≥ 40 U/L			33.0	96.4	82.5	73.6	74.8	
				Combo CDT–GGT ≥ 3.35			33.5	98.0	89.6	74.0	76.0	
				EtG ≥ 0.1 mg/L			16.9	100	100	70.0	71.7	

* Prediction characteristics evaluated in each study included sensitivity, specificity, NPV, PPV, accuracy, and AUC derived from ROC curves. Sensitivity refers to the probability that the test is positive when the condition is present. Specificity refers to the probability that the test is negative when the condition is not present. PPV refers to the probability that the condition is present when the test is positive. NPV refers to the probability that the condition is not present when the test is negative. Accuracy refers to the overall probability that the case is correctly classified from the test. Finally, AUC is derived from creating receiver operating curves by plotting the true positive rate (sensitivity) relative to the false positive rate (1-specificity). The area under the curve references the area on the graph created by the regression line relative to the chance rate of prediction. Values of 1 would indicate perfect condition and values of 0.50 would indicate chance prediction using a yes/no model. Predictive validity values are presented as percentages with the exception of AUC values, which are reported in proportions of accurate diagnostic classification with values of 0 to 1.00.

*Note:* ADHD, attention-deficit/hyperactivity disorder; ARBD, alcohol-related birth defects; ARND, alcohol-related neurodevelopmental disorder; AUC, under the curve; CDT, carbohydrate-deficient transferrin; EtG, ethyl glucuronide; FAEE, fatty acid ethyl esters; FAS, fetal alcohol syndrome; FASD, fetal alcohol syndrome disorders; GGT, gamma-glutamyltransferase; HC, head circumference; IGF-II, insulin-like growth factor-II; mg/l, milligrams per liter; mRNAs, messenger RNAs; nmol/g, nanomoles per gram; NPV, negative predictive value; PAE, prenatal alcohol exposure; PPV, positive predictive value; ROC, receiver operating characteristic; U/L, units per liter.

**Appendix 3:** Predictive Characteristics* of Other Screening Tools for Fetal Alcohol Spectrum Disorders and Its Associated Symptoms

Author	PAE/FASD Sample	Comparison Sample	Predictor	Outcome	Sensitivity	Specificity	PPV	NPV	Accuracy	AUC from ROC

Aring et al., 2021^66^	37 Children with FASD	65 Healthy children, 33 ADHD, 57 Moderate to late prematurity	FASD Eye Code ≥ 10	FASD	43	94				0.78
		65 Healthy children only			43	100				0.87
		16 Silver-Russell syndrome			43	88				0.6
		65 Healthy children								0.92
		All comparison groups								0.76
		33 ADHD								0.66
		57 Moderate to late prematurity								0.75
		65 Healthy children only	FASD Eye Code ≥ 9		57	98				0.87

Astley & Clarren, 1996^58^	42 FAS: 21 development sample and 21 validation sample	84 without FAS (including 4 with other genetic conditions) placed into 2 groups; 42 per group for development and then validation	Facial features: 2D continuous measurements philtrum/lip	Gestalt FAS	100	93				
			Likert scale rating philtrum & lip		100	100				
			Likert scale philtrum/lip continuous		100	100				

Astley et al., 2002^59^	Sampled 600 children in foster care screened	Facial analysis software	Facial features: 2D clinical FASD diagnosis	Screened + Gestalt FAS	100	99.8	85.7	100	99.8	

Moore et al., 2001^60^	41 FAS & 59 pFAS	31 Controls	Facial features: 6 2D craniofacial measurements	Clinical FAS/pFAS	98	90			96	
			2 Craniofacial measurements		100	100			100	
			5 Craniofacial measurements		86	94			88	

Widder et al., 2021^61^	22 FASD	31 Controls; 15 ADHD; 20 AUD/OUD; 18 depression	Facial features: 2D German BSI-FASD	Clinical FASD	77	70–100				
			Facial analysis software		67	44–79				
			German BSI-FASD adapted scoring		86	70–100				

Fang et al., 2008^62^	50 Finnish FASD diagnosis	32 Finnish controls	3D facial coordinates	FASD	88.2	100	100	83.3	92.6	
	36 FAS Cape-Colored	31 Finnish controls			91.7	90	91.7	90	90.9	
	86 Combined FASD	63 Combined controls			82.8	76.2	82.8	76.2	80	

Mutsvangwa et al., 2010^64^	4 FAS (age 5)	11 Controls (age 5)	3D facial coordinates	Clinical FASD	80	100	100	90.9	93.3	
	13 FAS (age 12)	6 Controls (age 12)			90.9	62.5	76.9	83.3	79	

Suttie et al., 2017^63^	22 FAS and 75 heavy AE South Africans (ages 6–18) & 35 FAS and 73 heavy AE Caucasians from CIFASD (ages 3–18)	69 South Africans (ages 6–18) who were Cape-Colored & 141 Caucasians from CIFASD (ages 3–18)	3D facial curvature coordinates of face	FAS or heavily AE						0.95–0.98
			3D facial curvature coordinates of profile							0.82–0.96
			3D facial curvature coordinates of eyes							0.92–0.95
			3D facial curvature coordinates malar							0.90–0.95
			3D facial curvature coordinates of mandible							0.85–0.93
			3D facial curvature coordinates of nose							0.86–0.95
			3D facial curvature coordinates of lip vermillion							0.69–0.84
			3D facial curvature coordinates of philtrum							0.70–0.90

Valentine et al., 2017^65^	36 FAS	50 Controls	Facial dysmorphology novel analysis technology computer scoring	FAS	78	92	88	85		0.95
	31 pFAS			pFAS	79	78	67	87		0.82
	22 ARND			ARND	50	92	70	83		0.84
	89 FASD			Any FASD	89	69	83	78		0.86
	36 FAS	50 Controls	Facial dysmorphology novel analysis technology manual scoring	FAS	99	89	87	99		0.96
	31 pFAS			pFAS	76	89	81	86		0.89
	22 ARND			ARND	43	92	70	79		0.74
	89 FASD			Any FASD	87	77	87	77		0.88

Jacobson et al., 2008^68^	12 FAS, 18 pFAS, 29 heavy PAE	20 Nonexposed controls; 4 nonexposed microcephalic	Physiological neural response: % criteria for eye-blink conditioning	FASD	70.2	75	87	51.4	71.6	
	10 FAS				100	75	62.5	100	82.4	

Kable et al., 2021^69^	26 PAE no diagnosis, 19 ARND, 5 FAS/pFAS	70 No PAE/no diagnosis	Physiological neural response: cardiac orienting response auditory COR Deviation Index	FASD						0.65
			Visual COR Deviation Index							0.77

Mesa et al., 2017^70^	Sample of Ukrainian infants with 26 having mild developmental delay	Sample of Ukrainian infants with 98 within normal limit development	Physiological neural response: cardiac orienting response Standard COR	12-month Bayley < 85			66	85		0.81
			Key features COR				62	82		0.81
			Maternal drinking				49	75		0.68
			Maternal drinking + standard COR				65	87		0.84
			Maternal drinking + key COR				62	80		0.8

Kaneko et al., 1996^71^	14 FAS	14 Controls	Physiological neural response: auditory event potentials	FAS	78.6	42.9	57.9	66.7	60.7	
		14 Down syndrome	Combination of P300 variables		78.6	85.7	84.6	80	82.1	

Tseng et al., 2013^78^	13 FASD	21 ADHD	19 Features of saccadic eye movements	FASD					90.4	
		18 Controls							79.2	
		21 ADHD and 18 controls			73	91			77.3	

Zhang et al., 2019^77^	91 FASD	116 Controls	Physiological neural response: eye tracking features of eye tracking, DTI, and neurobehavioral testing	FASD	81.8	87.5			84.8	
			Prosaccade						69.6	
			Antisaccade						76.1	
			Mesasaccade						65.2	
			DTI (4 features)						67.4	
			Neurobehavioral (3 domains)						78.3	

Bookstein et al., 2007^79^	23 PAE	21 Unexposed or lightly exposed	Neuroimage: MRI “hook” feature of corpus callosum	AE	52.2	95.2	92.3	64.5	72.7	

Little & Beaulieu, 2020^80^	79 FASD	81 Controls	Neuroimage: MRI 10 heavily weighted brain regions	FASD	64	88			77	

Burd et al., 1999^84^	1,013 Screened in school system with 6 FAS	1,007 Screened in school/no FAS diagnosis	Screening tool completed by trained staff > 20	Screened + FASD	100	94.1	9.2	100	94	

Burd et al., 2003^85^	152 FAS	IOM FAS cohort	FAS Diagnostic Checklist-total	FAS	84.9	82.4	75.4	89.5		
	157 pFAS			pFAS	54.3	83.3	66.1	74.1		
	87 PAE not FAS			PAE Not FAS	77	90.8	70.5	93.2		

Grant et al., 2013^86^	25 FASD (FAS, ARND, FAE, static encephalopathy)	463 No PAE	Self-report interview life history screen/addiction severity index	FASD	80.8	65.5			67.6	

Klug et al., 2021^81^	76 FASD	76 Controls	Caregiver Questionnaire: Checklist ARND-BC Parent All Questions	FASD	91.9	95.8	95.2	92.5		
			ARND-BC parent questions positive from binary regression		90.8	92.8	92.4	91.3		
			ARND-BC parent questions positive from continuous regression		89.7	94.5	94	90.5		
			ARND-BC parent questions sum of positive domains		89.7	94.5	94	90.5		

Nash et al., 2006^82^	54 FASD (ages 6–16)	30 Controls	Caregiver Responses to CBCL (7 items)	FASD				96.5		
		30 Controls	Caregiver responses to CBCL (6/7 items)		86	82				
		30 Controls	Caregiver responses to CBCL (5/7 items)		80	70	80	90.1		
		30 ADHD	Caregiver responses to CBCL (6/7 items)					86.3		
		30 ADHD	Caregiver responses to CBCL (3 items)		81	72				
		30 ADHD	Caregiver responses to CBCL (2 items)		70	80		84.9		

Nguyen et al., 2014^83^	79 PAE + ADHD; 36 PAE – ADHD	90 Controls + ADHD; 16 Controls – ADHD	Caregiver responses to BRIEF	AE					71.4	

Bernes et al., 2021^90^	177 Alcohol exposed CIFASD II	204 Controls CIFASD II	Low cutoff > 1.5 neurobehavioral battery & dysmorphology exam	AE	76.9	76.5	66	84.8	76.6	
			High cutoff > 2 neurobehavioral battery & dysmorphology exam	AE	63.6	87.8	75.5	80.3	78.8	
	177 Alcohol exposed CIFASD III	346 Controls CIFASD III	Low cutoff > 1.5 neurobehavioral battery & dysmorphology exam	AE	83.1	59	50.9	87.2	67.1	
			High cutoff > 2 neurobehavioral battery & dysmorphology exam	AE	66.1	77.5	60	81.7	73.6	
			Classification 3 AE vs. ADHD; latent profile analysis of complex neurobehavioral battery, dysmorphology, growth	AE	59.8	75.8	87.4	40	64	

Coles et al., 2020^91^	82 High risk ARND CoFASP sample of 1st graders	80 No risk CoFASP sample of 1st graders	Comprehensive neuropsychology battery	ARND CoFASP	74.4	83.8	82.4	76	79	
	85 Low-risk ARND CoFASP sample of 1st graders	73 No risk CoFASP sample of 1st graders			90.6	89	90.6	89	89.9	

Luca et al., 2016^87^	21 FAS	86 No FASD	Neurodevelopmental: quick screen neurological test-2	FAS	31.8	86.1	36.8	83.2	75	
	60 PAE	42 No PAE		AE	18.3	87.5	64.7	46.2	49.1	

Goh et al., 2016^88^	146 CIFASD II AE	288 No AE	Neurodevelopmental: complex neuropsychological battery	AE	74.2	89.9	78.6	87.4	84.6	
	55 CIFASD III child AE	110 No AE	Psychologist decision tree incorporating complex neurobehavioral battery & physical exam	AE	70.7	93.5	87.9	82.9	84.5	
	98 CIFASD III adolescent AE	191 No AE		AE	79.3	87.6	77.4	88.7	84.7	
	146 CIFASD II AE	288 No AE		AE	79.2	80.6	70.7	86.7	80.1	
	55 CIFASD III child AE	110 No AE		AE	63.8	93.4	85.7	80.7	82.1	
	98 CIFASD III adolescent AE	191 No AE		AE	81.3	78.3	71.4	86.2	79.5	

Johnston et al., 2019^92^	43 FASD	20 PAE but no FASD	Movement battery of tests (-2 SD, < 2nd percentile)	FASD	2–38	80–100				
			Movement battery of tests 5th percentile		9–75	68–100				
			Movement battery of tests 9th percentile		35–85	60–85				
			Movement battery of tests 16th percentile		44–83	45–70				

Mattson et al., 2010^93^	CIFASD children ages 8–17; 41 AE/FAS	46 CIFASD controls	Latent profile analysis of a complex battery	AE/FASD	87.8	95.7	94.7	89.8	92	
	CIFASD children ages 8–17; 41 alcohol-exposed/deferred not FAS	60 Controls	Latent profile analysis of a complex battery	AE/deferred FAS	68.4	95	89.7	82.6	84.7	

Mattson et al., 2013^89^	CIFASD children (ages 8–17) 209 AE (79 were FAS)	185 Controls; 74 ADHD	Neurodevelopmental: complex neuropsychological battery Latent profile analysis of complex neurobehavioral battery Dysmorphia, growth	AE/FASD	77.2	75.7	57.6	88.6	76.1	
			Classification 2 AE/non-FAS vs. controls	AE/FASD	70.1	72.4	61.7	79.3	71.5	

Thorne & Coggins, 2008;^94^ Thorne et al., 2007^95^	16 FASD	16 Normal controls	Narrative speech samples (NSS)-ANRTW	FASD	87.5	75			81.3	0.86
			NSS-PR		81.3	62.5			71.9	0.77
			NSS-ANR		81.3	81.3			81.3	0.76
			NSS-ANR Cutoff 1.7%						100	1
			NSS-AR							0.76
			NSS-Nominal Reference Errors (rNRE)						88	0.9
			NSS-Nominal Reference Errors (rNRE) 2%						97	0.98

* Prediction characteristics evaluated in each study included sensitivity, specificity, NPV, PPV, accuracy, and AUC derived from ROC curves. Sensitivity refers to the probability that the test is positive when the condition is present. Specificity refers to the probability that the test is negative when the condition is not present. PPV refers to the probability that the condition, is present when the test is positive. NPV refers to the probability that the condition is not present when the test is negative. Accuracy refers to the overall probability that the case is correctly classified from the test. Finally, AUC is derived from creating receiver operating curves by plotting the true positive rate (sensitivity) relative to the false positive rate (1-specificity). The AUC references the area on the graph created by the regression line relative to the chance rate of prediction. Values of 1 would indicate perfect condition, and values of 0.50 would indicate chance prediction using a yes/no model. Predictive validity values are presented as percentages with the exception of AUC values, which are reported in proportions of accurate diagnostic classification with values of 0 to 1.00. The different categories of predictive data (facial, neurophysiological, neuroimaging, questionnaire, and psychological performance measures are shaded from white to dark blue.

*Note*: 2D, two-dimensional; 3D, three-dimensional; ADHD, attention-deficit/hyperactivity disorder; AE, alcohol-exposed; ANRTW, Ambiguous Normal Reference Total Word; ARND, alcohol-related neurodevelopmental disorder; ARND-BC, Alcohol-Related Neurodevelopmental Disorder Behavior Checklist; AUC, area under the curve; AUD, alcohol use disorder; BRIEF, Behavior Rating Inventory of Executive Function; BSI-FASD, biographic screening interview for fetal alcohol spectrum disorders; CBCL, Child Behavior Checklist; CIFASD, Collaborative Initiative on Fetal Alcohol Spectrum Disorders; CIFASD-II, CIFASD, Phase II; CIFASD-III, CIFASD, Phase III; CoFASP, Collaboration on FASD Prevalence; COR, cardiac orienting response; DTI, diffusion tensor imaging; FAE, fetal alcohol effect; FAS, fetal alcohol syndrome; FASD, fetal alcohol syndrome disorders; IOM, Institute of Medicine; MRI, magnetic resonance imaging; NPV, negative predictive validity; NSS, narrative speech samples; NSS-ANR, narrative speech sample–ambiguous normal reference; NSS-AR, narrative speech sample–ambiguity rate; NSS-PR, narrative speech sample–pronoun reference; OUD, opioid use disorder; PAE, prenatal alcohol exposure; pFAS, partial fetal alcohol syndrome; PPV, positive predictive value; rNRE, rate of nominal reference errors; ROC, receiver operating characteristic; SD, standard deviation.
